# Linking nationwide health and social registry data to inform the policy for Tuberculosis contact tracing in Brazil.

**DOI:** 10.23889/ijpds.v7i3.2073

**Published:** 2022-08-25

**Authors:** Mauro N. Sanchez, Priscila F.P.S. Pinto, Helen Daniels, Camila S.S. Teixeira, Joilda S. Nery, Davide Rasella, Samila O.L. Silva, Maria Yury Ichihara, Mauricio L. Barreto, Julia M. Pescarini

**Affiliations:** 1 Department of Public Health, University of Brasilia, Brazil; 2 Centro de Integração de Dados e Conhecimentos para Saúde (Cidacs), Fundação Oswaldo Cruz, Salvador, Brazil; 3 Instituto de Saúde Coletiva, Universidade Federal da Bahia, Salvador, Brazil; 4 ISGlobal, Barcelona, Spain; 5 Faculty of Epidemiology and Population Health, London School of Hygiene & Tropical Medicine, London, United Kingdom

## Objectives

Mitigating the socioeconomic determinants of Tuberculosis (TB) and systematic screening of contacts and high-risk groups are targets of The World Health Organization (WHO) End TB Strategy by 2035. Our aim was to link socioeconomic information to TB datasets to inform policy makers in Brazil and contribute to addressing current challenges.

## Approach

Following a signed technical cooperation agreement with the Ministry of Health (MoH), we linked nationwide data on 1.405.682 registries of TB diagnosed between 2004 and 2019 in Brazil to 131.697.800 demographic and socioeconomic registries from the 100 Million Brazilian Cohort (2001-2018) previously linked to nationwide mortality data. We established close links with TB managers to understand the database, clean and deduplicate registries and to analyse the data. We took advantage of the data structure, to set up a cohort of household contacts of TB patients and produce estimates of TB incidence by subgroups of demographic and socioeconomic characteristics.

## Results

The interaction of the MoH was effective and facilitated by a robust TB Programme in the country. 567.999 (40,41%) TB cases were linked to the 100 Million Brazilian Cohort with high specificity (93.6%) and sensitivity (94.6%). Using family identifiers, we established the first TB case within a family unit (i.e., primary case) and followed their household contacts up to 15 years. We found the TB incidence among household contacts to be 427.8/100.000 person-years (95%CI 419.1-436.8). In the first year following the identification of the primary case, there was higher cumulative incidence among household contacts under 5 years of age, which was followed by a plateau of cases in this age group. Cummulative incidence in the other age groups presented a constant increase over time.

## Conclusion

The close collaboration with the MoH, the development of an effective linkage algorithm and the availability of large socioeconomic data allowed for a unique analysis of the high incidence of TB among household contacts. Findings reinforce need for constant dialogue among stakeholders to strengthen case detection by primary health care.

